# Performance of semiconductor dosimeters with a range of radiation qualities used for mammography: A calibration laboratory study

**DOI:** 10.1002/mp.14005

**Published:** 2020-01-20

**Authors:** Elisabeth Salomon, Peter Homolka, Istvan Csete, Paula Toroi

**Affiliations:** ^1^ Section of Dosimetry and Medical Radiation Physics International Atomic Energy Agency 1220 Vienna Austria; ^2^ Center for Medical Physics and Biomedical Engineering Medical University of Vienna 1090 Vienna Austria

**Keywords:** calibration, dosimetry, mammography, semiconductor dosimeter, radiation quality

## Abstract

**Purpose:**

To investigate the radiation quality dependence of the response of commercial semiconductor‐based dosimeters, and to estimate potential errors and uncertainties related to different measurement and calibration scenarios.

**Methods:**

All measurement results were compared to reference values measured at the IAEA dosimetry laboratory which is traceable to the international system of units (SI). Energy dependence of the response of eight semiconductor dosimeters were determined for five different anode‐filter combinations and tube voltages from 25 to 35 kV. For systems capable of deriving half value layer (HVL) and tube voltage from measurements, calibration coefficients for these measurements were calculated.

**Results:**

For six dosimeters, the maximum deviations from the reference value of the air kerma measurement were within ±5% as required by IEC 61674. Calibration coefficients for radiation qualities (anode‐filter and tube voltage combinations) relative to reference radiation quality Mo‐Mo 28 kV deviate up to 12%. HVL and tube voltage measurements exhibited deviations up to 11% and 10%, respectively.

**Conclusions:**

The air kerma responses of modern semiconductor dosimeters have a small energy dependence. However, no dosimeter tested complied with the accuracy limits stated by the manufacturer for tube voltage measurements, and only two dosimeters complied with the limits for HVL measurements. Absolute measurement of HVL and tube voltage with semiconductor dosimeters have to be verified for actual clinical radiation conditions on clinical mammography systems. Semiconductor dosimeters can be used for quality control measurements if individual calibration coefficients are available for the radiation condition applied. If other conditions are applied, additional uncertainty needs to be considered, particularly in the case of HVL and tube voltage measurements.

## 1 Introduction

Radiation dose to patients needs to be optimized to achieve both a reasonably low radiation burden to the patient and appropriate image quality to guarantee the necessary level of diagnostic confidence.^1^ Regular quality control of the x‐ray system is imperative,^2^, ^3^ including accurate measurements of radiation output and half‐value layer (HVL).

The quantity of interest in patient‐related dosimetry for mammography is the MGD (mean glandular dose). A direct measurement of MGD is impossible, therefore, tabulated factors are used to convert incident air kerma to MGD. These conversion factors are determined with Monte Carlo simulations and tabulated as a function of HVL and compressed breast thickness.^4^, ^5^, ^6^ Therefore, accurate air kerma and HVL measurements are needed.

Tube voltage measurements are stipulated for acceptance testing and on indication by both European and IAEA quality assurance guidelines.^2^, ^3^ European guidelines^2^ additionally recommend a tube voltage measurement every 6 months. Invasive measurement of tube voltage is the most accurate method but it is not practical for clinical use. Since most semiconductor dosimeters have the option of indicating tube voltage, these devices are an attractive solution for the user.

Measurements for quality control are performed either with ionization chambers or semiconductor‐based dosimeters. To ensure a satisfactory level of accuracy, regular calibration of the instrument is crucial.^1^, ^7^ Air kerma calibration of a field instrument is typically performed in a Secondary Standards Dosimetry Laboratory (SSDL). Radiation quality influences the response of ionization chambers and, to a much larger extent, semiconductor dosimeters.^8^, ^9^ However, the irradiation conditions in the calibration laboratory may differ considerably from clinical ones.

The range of clinically used radiation qualities is large and depends on the type of x‐ray equipment and protocol settings. The standardized radiation qualities for use in the determination of characteristics of x‐ray systems are specified in IEC 61267^10^ and they were also recommended for calibrations.^7^ However, these qualities do not cover the large variety of clinically used radiation qualities. In addition, it is not realistic for calibration laboratories to provide calibration services to cover all potential clinical situations.

The minimum requirements for a satisfactory level of performance for diagnostic dosimeters and standardized methods for the determination of compliance with these levels of performance are defined in IEC 61674.^11^ It defines limits of variation for the energy dependence of response for mammography dosimeters with ±5% for 25 to 35 kV. However, it does not give any limits for HVL nor tube voltage measurements.

## 2 Materials and Methods

### 2.A Protocol and reference standard

**Figure 1:**
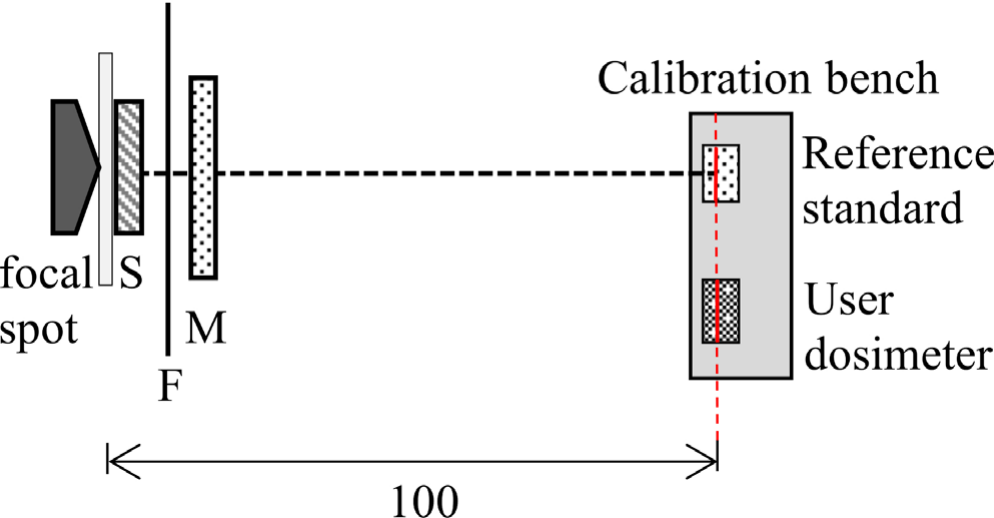
Calibration setup. S: Shutter, F: Filtration, M: Monitor chamber, Red dashed line: point of test and reference plane of the dosimeters. [Color figure can be viewed at http://wileyonlinelibrary.com]

The calibrations were performed according to IAEA TRS‐457 7 as described in the appendix of the IAEA calibration certificate,^12^ applying a substitution method using a monitor chamber for normalization of x‐ray beam output. A sketch of the calibration set up is shown in Fig. 1. The reference standard dosimeter at the IAEA dosimetry laboratory is an ionization chamber (Radcal 10X5‐6M, Radcal Cooperation, Monrovia, USA) connected to a Keithley 6517A (Keithley Instruments, Solon, USA) electrometer, being traceable to the primary laboratory at the PTB (Physikalisch‐Technische Bundesanstalt, Braunschweig, Germany) or BIPM (Bureau International des Poids et Mesures, Sèvres, France), depending on the radiation quality.

The variation in energy response of this chamber is not more than ±0.3% over the range of radiation qualities used in this study. It is much lower than stated in the specification sheet and stipulated by IAEA TRS‐457^7^ which recommends an ionization chamber with a maximum variation in the energy response of ±2.6%. For the calibrations performed in this study, a working standard was used. This working standard is an ionization chamber of the same type as the reference chamber and was calibrated against the reference standard dosimeter prior to measurements.

The mammographic x‐ray beam qualities with molybdenum and tungsten anodes were generated with x‐ray tube types RTW MCD 100H‐5Mo and Isovolt MXR160/0.4‐3.0, respectively. Air kerma rate was 50 mGy/min at 1 m distance to the focal spot.^12^ The output of the high‐voltage generator, type ISOVOLT160 Titan E, is monitored by a high‐voltage divider, type FUG HVT 160000, calibrated at PTB.

Purity and thickness measurements of the Al sheets used for the HVL determination comply with the recommendations in IAEA TRS‐457.^7^ Furthermore, a narrow beam, low scattering set‐up was applied for HVL measurement. In contrast to the sketch of the calibration set up shown in Fig. 1 for HVL measurements an additional beam limiting diaphragm was inserted at 0.5 m distance from the focal spot to achieve the desired geometry.

#### 2.A.1 Radiation qualities

In order to simulate clinically used radiation qualities, the selection was extended from standard radiation qualities^10^ to cover all together five anode‐filter combinations and four tube voltages. Radiation qualities and 1st HVL as well as the source of traceability of the working standard ionization chamber are summarized in Table 1.

**Table 1:** Characteristics of the radiation qualities chosen for this study.

Radiation quality	Filtration	1st HVL in mm Al				Source of traceability
		25 kV	28 kV	30 kV	35 kV	
Mo‐Mo	0.033 mm Mo	0.29	0.32	0.34	0.38	BIPM
Mo‐Rh	0.029 mm Rh	–	0.39	0.41	0.45	PTB
W‐Al	0.5 mm Al	0.31	0.35	0.38	0.44	PTB
W‐Rh	0.048 mm Rh	0.47	0.50	0.51	0.55	PTB
W‐Ag	0.049 mm Ag	0.48	0.53	0.56	0.61	PTB

### 2.B Dosimeters tested

Eight commonly used semiconductor dosimeters were calibrated. The dosimeters and the software used are listed in Table 2. The dosimeters require a preselection of the anode‐filter combination as well as the selection of compression paddle used (or not) before the measurement. Available selections and the quantities measured are listed in Table 3.

**Table 2:** Dosimeters calibrated in this study.

Manufacturer	Detector assembly	Measuring assembly	Software
RTI^a^	Internal detector	Piranha 657	Ocean, Version: 2010.12.15.38
RTI^a^	R100B^e^	Barracuda	Ocean, Version: 2010.12.15.38
Unfors^b^	Internal detector	Mult‐O‐Meter	Display read directly
RTI^a^	Internal detector	Black Piranha	Ocean, Version: 2016.06.14.214
PTW^c^	Internal detector	Nomex Multimeter	Nomex Software S030008, 3.0
Unfors^b^	MAM	Xi	RaySafe Xi View, Version: 3.0 (built 47)
Radcal^d^	AGMS–DM+	Accu Gold	Accu‐Gold by Radcal, Version 1.5.3.0 Driver Version 02.10.00
Unfors^b^	MAM	X2	RaySafe X2 View, Version 1.7.8.0

a Mölndal, Sweden.

b Billdal, Sweden.

c Freiburg, Germany.

d Monrovia, CA, USA.

e Not recommended by the manufacturer for mammography since no automatic compensation is available.

**Table 3:** Quantities measured and available selections of anode‐filter combinations in the software menu.

Measuring assembly	Indications			Calibration in the software				
	Dose rate	HVL	Tube voltage	W‐Al	W‐Rh	W‐Ag	Mo‐Rh	Mo‐Mo
Piranha 657	×	–	×	×	×	×	×	×
Barracuda	×	–	–	–	–	–	–	×
Mult‐O‐Meter	×	–	–	–	–	–	–	×
Black Piranha	×	×	×	×	×	×	×	×
Nomex	×	×	×	–	×	×	×	×
Xi	×	×	×	×	×	×	×	×
Accu Gold	×	×	×	–	×	×	×	×
X2	×	×	×	×	×	×	×	×

×, selection available; –, selection not available; HVL, half value layer.

#### 2.B.1 Determination of the calibration coefficient

The term calibration coefficient is used throughout this article regardless of the units. Generally, the calibration coefficient is determined as the ratio of the reference value and the value indicated by the instrument under calibration. The reference air kerma is calculated using the equation

(1) $${\dot{K}}_{\mathrm{air}}={\dot{M}}_{\mathrm{ref}}N_{\mathrm{ref}}k_{\mathrm{elec}}k_{\mathrm{TP}}$$

where ${\dot{M}}_{\mathrm{ref}}$ corresponds to measured ionization current, $N_{\mathrm{ref}}$ to the calibration coefficient of the working standard, *k_elec_* is a correction factor for the electrometer and *k_TP_* is a correction for the density of air.

The air kerma calibration coefficient *N_K_* is the ratio of the reference air kerma rate $\dot{K}$_air_ [*mGy/s*] obtained with the standard, and the reading $\dot{M}$_dos_ [*mGy/s*] of the user dosimeter:

(2) $$N_{K}=\frac{{\dot{K}}_{\mathrm{air}}\left[mGy/s\right]I_{\mathrm{dos}}}{{\dot{M}}_{\mathrm{dos}}\left[mGy/s\right]I_{\mathrm{ref}}}$$

The pressure and temperature corrected monitor chamber currents *I_ref_* and *I_dos_* for the reference and user dosimeters were used to account for potential changes in the tube output according to IAEA TRS‐457.^7^ Correspondingly, the calibration coefficients for HVL (*N_HVL_*) and tube voltage (*N_kV_*) were also calculated as the ratio of the reference value and the value indicated by the instrument under calibration.

#### 2.B.2 Uncertainties

Measurement uncertainties were estimated according to^13^ and^14^ and classified as type A or type B. Type A uncertainties are evaluated by statistical analysis of the results of a series of observations. Type B uncertainties are determined by other means.

The uncertainty of the calibration coefficient consists of uncertainties related to reference air kerma measurement and measurements performed with the user dosimeter. The major component of uncertainty is the calibration coefficient of the working standard which was estimated according to the appendix of the calibration certificate.^12^ This uncertainty (k = 2) is 1.0% or 1.3% depending on the traceability. However, for consistency the higher value was used for all uncertainty estimations. The uncertainty related to measurements performed with the user dosimeters were individually estimated and a conservative estimate was used for the uncertainty budget given in Table 4.

**Table 4:** Estimated relative uncertainty in percent for of the calibration of the semiconductor dosimeters against the working standard.

		Type A	Type B
Step 1: Working standard			
Calibration against the reference standard		0.04	0.63
Chamber positioning			0.03
Current measurements including range and time‐based corrections of IAEA electrometer		0.02	0.05
Uncertainty due to temperature measurements		0.01	0.03
Uncertainty due to pressure measurements		0.01	0.01
Monitor chamber contribution^a^		0.02	
Relative combined standard uncertainty of ${\dot{K}}_{\text{air}}$ (Step 1)		0.05	0.63
Step 2: Semiconductor dosimeter			
Positioning			0.03
Reading and resolution			0.12
Relative combined standard uncertainty of ${\dot{M}}_{\mathrm{dos}}$ (Step 2)			0.12
Relative combined standard uncertainty of ${\dot{N}}_{K}$ (Step 1 + 2)	0.05		0.65
Expanded uncertainty (k = 2)	1.3		

a Includes uncorrelated part of uncertainties related to reading, leakage and short‐ term stability of the monitor chamber in both measurements with the reference and user dosimeter.

The expanded (k = 2) relative uncertainties of the first HVL values and the adjusted direct current high voltages of the x‐ray tubes were estimated. The major components of the uncertainty of reference HVL are thickness of the HVL filters, change in the spectra and interpolation, resulting in an uncertainty of not more than 1%. The uncertainty of the reference tube voltage values of less 1% results mainly from the calibration of the high voltage divider.

## 3 Results

### 3.A Calibration coefficients of air kerma rate

**Figure 2:**
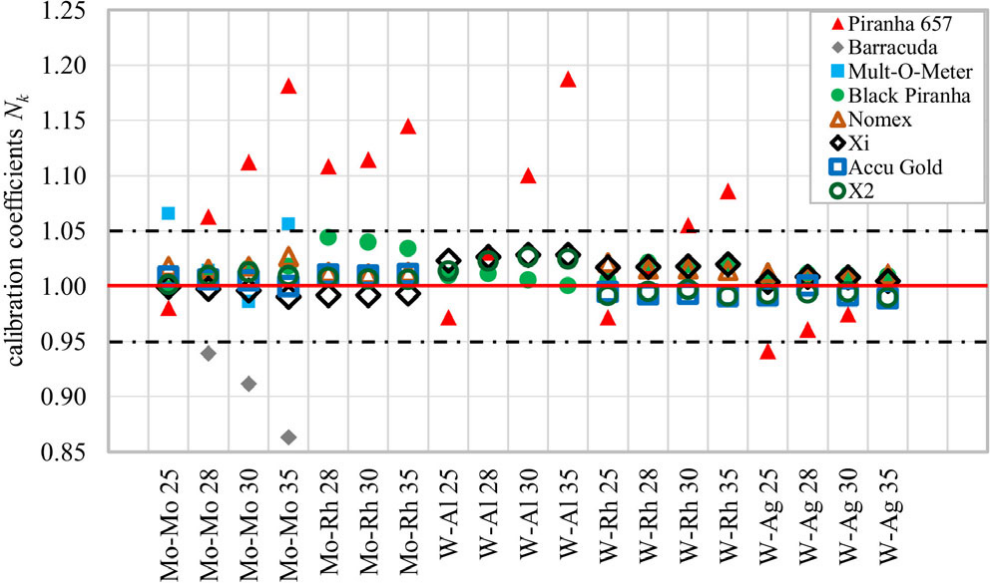
Air kerma calibration coefficients *N_k_* for all semiconductor dosimeters tested as a function of radiation quality. The horizontal red line indicates the ideal calibration coefficient of 1.0, the dashed black lines represent ±5% tolerance level. Piranha 657, Barracuda, Mult‐O‐Meter, Black Piranha, Nomex, Xi, Accu Gold, X2. [Color figure can be viewed at http://wileyonlinelibrary.com]

Eight semiconductor dosimeters were calibrated in a calibration laboratory to investigate the impact of radiation quality on the response. Calibration coefficients *N_k_* as a function of radiation qualities for the semiconductor dosimeters tested are shown in Fig. 2. The horizontal red line represents the ideal calibration coefficients of one, the dashed black lines represent ±5% tolerance level. Expanded uncertainty for the calibration coefficient of air kerma rate measurement for all dosimeters is <1.3%. This uncertainty estimation does not include long‐term reproducibility. The individual calibration coefficients are provided in the appendix (Table S1).

### 3.B Calibration coefficients for half‐value layer measurements

Five of the dosimeters tested derive HVL values from measurements. The maximum standard deviation in short term repeatability was ±0.007 mm Al. The specified accuracy of the HVL measurement was stated by each manufacturer in a different way. Accu Gold did not provide a reading for W‐Ag 35. The manufacturers’ specifications and the maximum deviations are listed in Table 5. All manufacturers stated the limits within a confidence interval of 95%. Detailed data are shown in the appendix (Table S2).

**Table 5:** Maximum deviation in percent of the half value layer measurements.

Measuring assembly	Manufacturer specification	Max deviation
Black Piranha	±10%	11% (0.04 mm Al)
Nomex	±0.01 mm Al	6.7% (0.04 mm Al)
Xi	±5%	11% (0.05 mm Al)
Accu Gold	±10% (0.05 mm Al)	−1.6% (−0.01 mmAl)
X2	±5%	−3.7% (0.01 mmAl)

### 3.C Calibration coefficients for tube voltage measurements

Six of the dosimeters tested derive tube voltage from measurements. The maximum standard deviation for short‐term repeatability was ±0.1 kV. As stated in the manual, Xi and X2 require additional 2 mm Al filtration for tube voltage measurements for Mo‐Rh. X2 did not provide a result for Mo‐Rh. Without additional filtration of 2 mm Al, the air kerma rate at the point of test was 0.83 mGy/s. The additional filtration reduces the air kerma rate to approximately 0.06 mGy/s. With increased air kerma rate (approximately above 0.1 mGy/s), measurement results for Mo‐Rh 30 and Mo‐Rh 35 could be obtained with X2 but not for Mo‐Rh 28. Xi did not measure tube voltage for the radiation quality W‐Ag. The manufacturers’ specifications as well as the maximum deviations are shown in Table 6. All manufacturers stated the limits within a confidence interval of 95%. Detailed data are shown in the appendix (Table S3).

**Table 6:** Maximum deviation in percent of tube voltage measurements.

Measuring assembly	Manufacturer specification	Max deviation
Piranha 657	±2% or ±1 kV	10% (2.8 kV)
Black Piranha	±2% or ±1 kV	10% (3.5 kV)
Nomex	±0.5 kV	8.2% (2.4 kV)
Xi	±2% or ±0.5 kV	7.1% (2.5 kV)
Accu Gold	±2% or ±0.7 kV	8.2% (2.3 kV)
X2	±2% or ±0.5 kV	−5.9% (2.2 kV)

## 4 Discussion

### 4.A Air kerma measurement

The impact of the radiation quality on the response of eight semiconductor dosimeters was investigated in a calibration laboratory. The selection of the tested dosimeters was made based on the limited information from the calibration laboratories in order to cover some of the most common models. This selection is comparable with the study of Brateman and Heintz^15^ and includes dosimeters that have been marketed for a long time as well as more recent models.

Table 7 shows the maximum errors occurring if the dosimeter reading is directly used without correction (no calibration) and multiplying the dosimeter reading by the calibration coefficients for the radiation quality Mo‐Mo 28 (single point calibration). The “no calibration” column simulates a situation where no calibration coefficient is applied, and the reading of the dosimeter is used without any additional correction, that is, the manufacturer's conversion is assumed to be correct. The “single point calibration” represents the situation when the dosimeter is calibrated only with one standard radiation quality. Three dosimeters exceeded the ±5% limit for air kerma measurement using manufacturer settings. Applying a single calibration coefficient for Mo‐Mo 28, the errors were clearly reduced but still the same three dosimeters exceeded the ±5% limit.

**Table 7:** Deviation in air kerma rate (error) in case no calibration or one single point calibration coefficient applied.

Measuring assembly	Maximum deviation	
	No calibration	Single point calibration with Mo‐Mo 28
Piranha 657	−16%	−12%
Barracuda	16%	8.1%
Mult‐O‐Meter	−6.1%	−5.1%
Black Piranha	−4.2%	−3.2%
Nomex	−2.6%	−1.1%
Xi	−2.8%	−3.1%
Accu Gold	1.1%	1.7%
X2	−2.5%	1.9%

Five dosimeters comply with the ±5% maximum limit of variation.^11^ However, if enhanced accuracy for the measurement is favored, the uncertainty related to energy dependence should be smaller as described in the IAEA TRS‐457.^7^ Only one dosimeter had lower than 1.5% variation of the response and could be considered to comply with reference level measurement without use of radiation quality specific calibration coefficients. None of the dosimeters could achieve lower than 1% energy dependence which is typically achieved with ionization chambers (table VIII.2 in Ref. [7]).

The energy dependence of the response is different for each dosimeter and no trend could be observed for the different anode filter combinations. Thus, interpolation based on the actual HVL might be an option for different tube voltages but not between different anode‐filter combinations.

### 4.B Measurement of HVL

In this study, only two dosimeters fully met their specified accuracy limits for HVL measurement. Only one dosimeter complied with +/‐3% limit stated in the IAEA TRS‐457 (table VIII.12 in Ref. [7]). Inaccurate HVL has a direct impact on the calculation of the MGD. The correction factors required to convert the incident air kerma to MGD are tabulated as a function of HVL, breast thickness, and glandularity in Ref. [4, 5, 6]. A difference of 8% in MGD calculation can occur for an error of 11% in HVL measurements as found in this study, assuming a 5 cm breast with 50% glandularity.

Most modern dosimeters have the option to select the anode‐filter combination even for a specific manufacturer to account for spectral differences between different systems. These settings cannot be fully tested in a calibration laboratory. They have to be calibrated *in situ* using those specific systems employing an ionization chamber with flat energy response and proper calibration. Verified semiconductor dosimeters could be used for quality control measurements if their long‐term stability is confirmed.

### 4.C Measurement of tube voltage

No dosimeter fully met the manufacturers' specifications for indicated tube voltage and the maximum deviations were 5.9% to 10%. The largest deviations occurred for radiation qualities with Mo‐anode. None of the dosimeters complied with the 2% limit which was stated as an uncertainty limit for this quantity by most of the manufacturers.

Xi did not provide a result for tube voltage measurement for the anode‐filter combination W‐Ag and X2 for Mo‐Rh. With increased dose rate, X2 measured the tube voltage for Mo‐Rh 30 and 35, but still failed for Mo‐Rh 28. The performance of tube voltage measurement of semiconductor dosimeters on clinical mammography units was investigated by Brateman and Heintz.^15^ There, Xi also failed to derive tube voltage from measurements for W‐Ag on the clinical mammography units. In that scenario, the dosimeter showed the same malfunction in the calibration laboratory and with measurements on the clinical mammography unit.

In general, absolute values of tube voltage are most accurately determined with invasive measurements but in most clinical situations this is not feasible and semiconductor dosimeters are used for this purpose. Based on this study such an approach can lead to errors without appropriate calibration. However, often in quality control checks, relative change of beam quality is more important parameter than the absolute value. For this purpose, semiconductor dosimeters might be useful, if their long‐term stability is measured and verified.

### 4.D The role of medical physicists

These results indicate the importance of a medical physicist being involved in the quality control and system testing process and being aware of limitations and specialties of the used equipment. This is crucial to ensure that all used dosimeters are suitable for the purpose. Reliance on the manufacturers' information alone is not sufficient. With a calibrated ionization chamber available, the medical physicist can verify the readings provided by the semiconductor dosimeter and also assess the long‐term stability. If possible, dosimeters should be calibrated for all radiation qualities and quantities of interest for which they are intended to be used. However, normally it is not possible to cover all the clinically used radiation qualities in the calibration laboratories. Therefore, a calibration procedure using standardized radiation qualities and a simplified procedure to check the performance of semiconductor dosimeters with the other radiation qualities should be developed. Further studies are required to investigate their performance in a radiation‐setup generated by a clinical mammography unit as well as the long‐term performance of the semiconductor dosimeters.

## 5 Conclusions

The performance of eight semiconductor dosimeters with five different anode‐filter combination was tested in the IAEA calibration laboratory. The energy dependence of the air kerma response was different for each dosimeter and varied from −16% to +16%. There was not a clear trend in the responses and therefore interpolation between different anode‐filter combinations is not recommended. Five dosimeters complied with the ±5% limit stated by IEC 61674^11^ but none of them reached the level ±1% which is typically achieved with ionization chambers (table VIII.2 in IAEA TRS‐457^7^).

Half value layer is a key parameter for MGD calculation. Only one dosimeter complied with the ±3% limit stated in IAEA TRS‐457.^7^ An error of 11% in HVL determination corresponding to the maximum deviation found in this study results in errors up to 8% in MGD calculations.

Tube voltage measurement with semiconductor dosimeters is especially challenging since no dosimeter fully complied with the specified accuracy limits and none of them achieved the limit ±2% as stated by most manufacturers. Therefore, the user should carefully consider if the related uncertainties and potential errors are acceptable for their purposes.

## Conflict of Interest

The authors have no conflict to disclose.

## Supporting information


**Table S1:** Calibration coefficients for air kerma rate measurements.
**Table S2:** Calibration coefficients for half value layer measurements.
**Table S3:** Calibration coefficients for tube voltage measurements.
