# Supplementary material for: Performance of semiconductor dosimeters with a range of radiation qualities used for mammography: A calibration laboratory study
Source: Med Phys. 2020 Jan 20;47(3):1372–8. doi: 10.1002/mp.14005 (PMC7079095; doi:10.1002/mp.14005)
Supplement: Supplementary file 1 — Table S1: Calibration coefficients for air kerma rate measurements. Table S2: Calibration coefficients for half value layer measurements. Table S3: Calibration coefficients for tube voltage measurements. [file MP-47-1372-s001.doc]

## APPENDIX

The calibration coefficients for air kerma rate, HVL and tube voltage measurements are listed in Table A 1,Table A 2 and Table A 3. Raw data are available from the corresponding author upon reasonable request.

| Table A 1:Calibration coefficients for air kerma rate measurements. | | | | | | | | |
| --- | --- | --- | --- | --- | --- | --- | --- | --- |
|  | **Piranha 657** | **Barracuda** | **Mult-O-Meter** | **Black Piranha** | **Nomex** | **Xi** | **Accu Gold** | **X2** |
| **Mo-Mo 25** | 0.98 | 0.99 | 1.07 | 1.00 | 1.02 | 1.00 | 1.01 | 1.00 |
| **Mo-Mo 28** | 1.06 | 0.94 | 1.01 | 1.01 | 1.02 | 1.00 | 1.01 | 1.01 |
| **Mo-Mo 30** | 1.11 | 0.91 | 0.99 | 1.02 | 1.02 | 1.00 | 1.01 | 1.01 |
| **Mo-Mo 35** | 1.18 | 0.86 | 1.06 | 1.02 | 1.03 | 0.99 | 1.00 | 1.01 |
| **Mo-Rh 28** | 1.11 | – | – | 1.04 | 1.01 | 1.00 | 1.01 | 1.01 |
| **Mo-Rh 30** | 1.11 | – | – | 1.04 | 1.01 | 0.99 | 1.01 | 1.01 |
| **Mo-Rh 35** | 1.14 | – | – | 1.03 | 1.01 | 0.99 | 1.01 | 1.01 |
| **W-Al 25** | 0.97 | – | – | 1.01 | – | 1.02 | – | 1.01 |
| **W-Al 28** | 1.03 | – | – | 1.01 | – | 1.03 | – | 1.02 |
| **W-Al 30** | 1.10 | – | – | 1.01 | – | 1.03 | – | 1.03 |
| **W-Al 35** | 1.19 | – | – | 1.00 | – | 1.03 | – | 1.02 |
| **W-Rh 25** | 0.97 | – | – | 1.01 | 1.02 | 1.02 | 0.99 | 0.99 |
| **W-Rh 28** | 1.02 | – | – | 1.02 | 1.02 | 1.02 | 0.99 | 0.99 |
| **W-Rh 30** | 1.06 | – | – | 1.02 | 1.02 | 1.02 | 0.99 | 1.00 |
| **W-Rh 35** | 1.09 | – | – | 1.02 | 1.01 | 1.02 | 0.99 | 0.99 |
| **W-Ag 25** | 0.94 | – | – | 1.00 | 1.01 | 1.00 | 0.99 | 0.99 |
| **W-Ag 28** | 0.96 | – | – | 1.01 | 1.01 | 1.01 | 1.00 | 0.99 |
| **W-Ag 30** | 0.97 | – | – | 1.01 | 1.01 | 1.01 | 0.99 | 0.99 |
| **W-Ag 35** | 1.01 | – | – | 1.01 | 1.01 | 1.00 | 0.99 | 0.99 |

| Table A 2: Calibration coefficients for HVL measurements. | | | | | | |
| --- | --- | --- | --- | --- | --- | --- |
|  | **Black Piranha** | **Nomex** | **Xi** | **Accu Gold** | **X2** |  |
| **Mo-Mo 25** | 0.90 | 0.95 | 0.96 | 0.99 | 0.97 |  |
| **Mo-Mo 28** | 0.91 | 0.95 | 0.97 | 1.00 | 0.98 |  |
| **Mo-Mo 30** | 0.92 | 0.95 | 0.97 | 1.00 | 0.99 |  |
| **Mo-Mo 35** | 0.93 | 0.96 | 0.96 | 1.00 | 1.01 |  |
| **Mo-Rh 28** | 0.91 | 0.97 | 0.97 | 1.00 | 0.99 |  |
| **Mo-Rh 30** | 0.92 | 0.98 | 0.97 | 0.99 | 0.99 |  |
| **Mo-Rh 35** | 0.94 | 1.00 | 0.97 | 0.99 | 0.99 |  |
| **W-Al 25** | 0.98 | – | 0.96 | – | 1.04 |  |
| **W-Al 28** | 1.01 | – | 0.97 | – | 1.03 |  |
| **W-Al 30** | 1.01 | – | 0.96 | – | 1.02 |  |
| **W-Al 35** | 1.00 | – | 0.90 | – | 1.00 |  |
| **W-Rh 25** | 1.03 | 0.99 | 0.96 | 1.02 | 1.01 |  |
| **W-Rh 28** | 1.01 | 0.94 | 0.95 | 0.99 | 0.99 |  |
| **W-Rh 30** | 0.96 | 0.94 | 0.96 | 0.99 | 0.99 |  |
| **W-Rh 35** | 0.96 | 0.94 | 0.96 | 1.00 | 1.00 |  |
| **W-Ag 25** | 1.00 | 1.04 | 0.99 | 1.00 | 1.00 |  |
| **W-Ag 28** | 0.99 | 1.01 | 0.98 | 1.01 | 0.99 |  |
| **W-Ag 30** | 0.99 | 1.01 | 0.98 | 1.00 | 0.99 |  |
| **W-Ag 35** | 0.98 | 1.00 | 0.97 | – | 0.99 |  |

| Table A 3: Calibration coefficients for tube voltage measurements. | | | | | | | |  |
| --- | --- | --- | --- | --- | --- | --- | --- | --- |
|  | **Piranha 657** | **Black Piranha** | **Nomex** | **Xi** | **Accu Gold** | **X2** |  | |
| **Mo-Mo 25** | 0.98 | 0.98 | 0.97 | 0.97 | 1.00 | 0.97 |  | |
| **Mo-Mo 28** | 0.96 | 0.96 | 0.96 | 0.96 | 1.00 | 0.96 |  | |
| **Mo-Mo 30** | 0.95 | 0.95 | 0.95 | 0.95 | 0.99 | 0.96 |  | |
| **Mo-Mo 35** | 0.94 | 0.94 | 0.94 | 0.93 | 0.99 | 0.94 |  | |
| **Mo-Rh 28** | 0.91 | 0.91 | 0.92 | 0.95 ^a^ | 0.92 | – |  | |
| **Mo-Rh 30** | 0.93 | 0.91 | 0.93 | 0.95 ^a^ | 0.93 | – |  | |
| **Mo-Rh 35** | 0.93 | 0.91 | 0.94 | 0.95 ^a^ | 0.94 | – |  | |
| **W-Al 25** | 0.97 | 0.99 | – | 0.99 | – | 0.99 |  | |
| **W-Al 28** | 0.97 | 0.99 | – | 0.99 | – | 0.98 |  | |
| **W-Al 30** | 0.97 | 0.99 | – | 0.98 | – | 0.98 |  | |
| **W-Al 35** | 0.97 | 0.99 | – | 0.97 | – | 0.97 |  | |
| **W-Rh 25** | 1.00 | 0.99 | 1.00 | 1.00 | 0.96 | 0.99 |  | |
| **W-Rh 28** | 1.01 | 0.98 | 0.99 | 0.98 | 0.97 | 0.98 |  | |
| **W-Rh 30** | 1.00 | 0.97 | 0.97 | 0.98 | 0.97 | 0.97 |  | |
| **W-Rh 35** | 0.99 | 0.97 | 0.97 | 0.98 | 0.97 | 0.96 |  | |
| **W-Ag 25** | 0.97 | 0.98 | 0.98 | – | 0.98 | 0.98 |  | |
| **W-Ag 28** | 0.99 | 0.98 | 0.97 | – | 0.96 | 0.99 |  | |
| **W-Ag 30** | 1.00 | 0.98 | 0.97 | – | 0.96 | 0.98 |  | |
| **W-Ag 35** | 0.99 | 0.96 | 0.96 | – | 0.96 | 0.97 |  | |
| ^a^ additional filtration of 2 mm Al as required by the manufacturer. | | | | | | |  | |
